# An Effective Ensemble Machine Learning Approach to Classify Breast Cancer Based on Feature Selection and Lesion Segmentation Using Preprocessed Mammograms

**DOI:** 10.3390/biology11111654

**Published:** 2022-11-11

**Authors:** A. K. M. Rakibul Haque Rafid, Sami Azam, Sidratul Montaha, Asif Karim, Kayes Uddin Fahim, Md. Zahid Hasan

**Affiliations:** 1 Health Informatics Research Laboratory (HIRL), Department of Computer Science and Engineering, Daffodil International University, Dhaka 1341, Bangladesh; 2 College of Engineering, IT and Environment, Charles Darwin University, Darwin, NT 0909, Australia

**Keywords:** breast cancer, classification, mammogram, segmentation, image processing, machine learning, feature extraction, ensemble model, feature selection, CBIS-DDSM

## Abstract

**Simple Summary:**

The screening of breast cancer in its earlier stages can play a crucial role in minimizing mortality rate by enabling clinicians to administer timely treatments and preventing the cancer from reaching the critical stage. With this view, the objective of this research is to develop an efficient automated approach for analyzing and classifying mammograms into four classes. Primarily, artefacts present in the mammograms are eliminated and the mammograms are enhanced utilizing image-processing techniques. When applying seven data augmentation methods, the volume of the mammography dataset is enlarged. Afterward, the region of interest (ROI) is extracted from the mammograms employing a region-growing algorithm with a dynamic intensity threshold calculated for each mammogram. From each ROI, a total of 16 geometrical features are extracted. These features are investigated with eleven state-of-the-art machine learning (ML) algorithms and depending on test accuracies, three ensemble models are developed. Among the ensemble models, the highest test accuracy of 96.03% is gained by stacking Random Forest and XGB classifier (RF-XGB). Furthermore, the performance of RF-XGB is boosted by utilizing various feature selection methods resulting in 98.05% accuracy. Moreover, the performance consistency of the best model is evaluated with the K-fold cross-validation experiment. This proposed approach of classifying mammograms may assist specialists in the precise and effective diagnosis of breast cancer.

**Abstract:**

Background: Breast cancer, behind skin cancer, is the second most frequent malignancy among women, initiated by an unregulated cell division in breast tissues. Although early mammogram screening and treatment result in decreased mortality, differentiating cancer cells from surrounding tissues are often fallible, resulting in fallacious diagnosis. Method: The mammography dataset is used to categorize breast cancer into four classes with low computational complexity, introducing a feature extraction-based approach with machine learning (ML) algorithms. After artefact removal and the preprocessing of the mammograms, the dataset is augmented with seven augmentation techniques. The region of interest (ROI) is extracted by employing several algorithms including a dynamic thresholding method. Sixteen geometrical features are extracted from the ROI while eleven ML algorithms are investigated with these features. Three ensemble models are generated from these ML models employing the stacking method where the first ensemble model is built by stacking ML models with an accuracy of over 90% and the accuracy thresholds for generating the rest of the ensemble models are >95% and >96. Five feature selection methods with fourteen configurations are applied to notch up the performance. Results: The Random Forest Importance algorithm, with a threshold of 0.045, produces 10 features that acquired the highest performance with 98.05% test accuracy by stacking Random Forest and XGB classifier, having a higher than >96% accuracy. Furthermore, with K-fold cross-validation, consistent performance is observed across all K values ranging from 3–30. Moreover, the proposed strategy combining image processing, feature extraction and ML has a proven high accuracy in classifying breast cancer.

## 1. Introduction

An unusual growth of breast tissues leads to breast cancer, which can cause uncontrolled cell division and formulation of mass which eventually spread to the other cells of the body. As mentioned above, one of the major killers is breast cancer which is increasing rapidly not only in developed but also in developing countries [1]. As of the end of 2020, 7.8 million women has been diagnosed as a victim of breast cancer in the past five years, which makes it the most prevalent disease worldwide [2,3]. Malignant tumors are typically classified as positive in clinical terms, while benign tumors are classified as negative [4]. According to a survey by WHO [3], every year, nearly one million women are newly spotted with breast cancer, and almost half of them pass away due to the delay in detection and treatment [3]. Such a high mortality rate can be prevented through early detection. However, breast cancer detection is difficult in distant locations due to the lack of high-quality medical resources, particularly highly experienced doctors [1]. Moreover, it is very difficult for clinicians to deal with the rapidly increasing number of positive cases. Mammography, nuclear magnetic resonance imaging, computed tomography technology, microwave imaging, photoacoustic imaging and other techniques are currently utilized to identify breast cancer. Among them, Mammography is considered a highly efficient method for identifying the cancer type [5]. It is often challenging for clinical experts to make a precise diagnosis based on mammography because of the intricacy of early breast cancer mammogram images, as well as the poor contrast of the mammogram images themselves. As a result, adopting a machine learning-based Computer-Aided Diagnosis (CAD) system, with lower computational complexity, can help clinicians by improving diagnostic accuracy [4]. In order to determine breast cancer, a radiologist has to determine the cancer region (ROI) which consists of calcifications in breast tissue. In most mammograms, the cancerous region appears as having a near similar intensity level with dense breast tissues which might lead to the interpretation of mammograms being a bit challenging. In this regard, our proposed approach can be highly beneficial as it detects the ROI (cancerous region) and segments them from the mammogram. This way, the radiologists do not need to go through the entire mammogram and can focus on only the cancerous part. In addition, size, pattern, area of the ROI and density of masses information are taken into consideration while diagnosing. In earlier stages, the breast cancer shows up as white dots, as breast cancer progresses, the calcification spreads and gets bigger in later stages. In clinical implementation, while diagnosing cancer, radiologists come to a decision by considering these structural alterations or distortions of the features of tumor and examining them. This study works by extracting various features from the cancerous region in an automated approach, which can significantly aid radiologists by giving a broad insight regarding the structural alterations in determining the stage of breast cancer. In this research, the CBIS-DDSM dataset is utilized to classify breast cancer, which is collected from the Kaggle repository containing 1459 mammograms of four classes named Malignant Calcification (MC), Malignant Mass (MM), Benign Calcification (BC) and Benign Mass (BM). As the mammogram images contain several artefacts, noises and low contrast levels, the dataset is preprocessed by employing several image processing algorithms. Moreover, it is discovered that the dataset contains a limited number of images, which are addressed using several augmentation techniques. The cancerous lesion is segmented by employing a dynamic approach based on the intensity level of each image and region-growing algorithm. Afterwards, 16 geometrical features are extracted from this ROI and a total of eleven ML algorithms are explored using the features. Three ensemble models are developed from these eleven algorithms using three different cases depending on test accuracy. The first ensemble model is built by stacking the ML models with an accuracy of above 90%, the second model is built by stacking the models with an accuracy of above 95% and the third model is built by stacking the models with an accuracy of above 96%. The best ensemble model is selected after training the three ensemble models again with the dataset. The performance is enhanced further by utilizing various feature selection methods. The robustness of the model is assessed by employing a cross-validation technique named K-fold where satisfactory performance is achieved across all the K-folds. The performance of our study in identifying breast cancer from mammograms is compared to other recent research done on a similar dataset. The approach can be highly effective in assisting radiologists in several clinical insights including reducing strain on doctors, detection at an early stage, saving time and less error.

## 2. Research Aim and Scope

The proposed automated system follows the path of a radiologist in screening mammograms. In the real screening realm of breast screening, a radiologist mainly focuses on the cancerous region and examines different features of that area in order to reach a decision. In this study, the same objective is carried out in an automated way, including segmenting cancerous regions, extracting features from them and, finally, providing a high-accuracy classification result using the features of the machine learning approach. However, if the mammograms are taken using a different protocol and scheme, the process of extracting ROI might be changed a little due to the appearance of different structures or intensity levels. In large-scale screening trials, it can be anticipated that the model will perform with the optimal outcome, requiring less time as it entirely follows the process of a clinical diagnosis. Moreover, an automated approach tends to perform even better while using a larger dataset. The following is a summary of the goals and methods:

- To begin with, several widely-used image-processing techniques, named Binary Masking, Largest Contour Detection, Canny Edge detection and Hough Lines Transformation, are employed successfully to remove the artefacts and afterwards, Gamma Correction and Contrast Limited Adaptive Histogram Equalization (CLAHE) are employed to enhance the brightness and contrast level of the mammograms.
- The volume of the dataset is increased from 1459 to 11,536 images by performing various augmentations methods.
- The region of interest (ROI) is retrieved from the preprocessed augmented mammograms by the help of a region-growing method where a dynamic intensity thresholding process is introduced.
- A total of 16 geometrical features are uprooted from these ROI images.
- A total of eleven ML algorithms named Decision Tree, Random Forest, Logistic Regression, AdaBoost, Support Vector classification, K Nearest Neighbors, Multilayer Perceptron, Gaussian Naive Bayes, Stochastic Gradient Descent, XG Boost and Support Vector Machine are applied to the geometrical features and three ensemble models are developed from the eleven models, depending on three thresholds derived from test accuracy.
- These ensemble methods are again trained with the extracted geometrical features and the ideal model is determined based on the highest accuracy.
- For enhancing the performance of the best model, five feature selection approaches named Random Forest feature importance (RF), Univariate features, Correlation Matrix, Principal Component Analysis method (PCA) and Wrapper Method are carried out with fourteen different configurations.
- The robustness of the best model is evaluated further by training the model and applying K-fold cross-validations with 12 K values beginning from 3 to 30.

## 3. Literature Review

Early Machine learning is often utilized by many researchers to identify the stages of breast lesions. Xuejiao et al. [6] applied discrete wavelet transform and the Fourier cosine transform methods to extract statistical features from mammogram images in order to classify breast cancer. An entropy-based technique was employed to find the optimal features. Experimenting with different classifiers, they obtained a maximum accuracy of 96.06% from the voting classification method. The authors of this study [7], presented a feature extraction-based classification model using Hough transform. After extracting the features, the SVM classifier was utilized for the classification of tumors into three classes of normal, benign and malignant. They achieved the highest accuracy of 95%. However, only 322 mammograms are used in this study and no augmentation technique is introduced. Moreover, the authors did not describe clearly the preprocessing methods used in this study. Mohamed et al. [8] proposed a feature extraction method employing the statistical t-test technique. The optimal number of features was selected in terms of the highest accuracy using a dynamic threshold scheme. SVM was applied as a binary classifier to categorize mammograms into benign and malignant and the highest accuracy of 95.98% was achieved. However, 322 mammograms were used in this study with no implementation of data augmentation. Their accuracy might be improved if image-processing techniques and some other classifiers would be explored. In this study [9], a feature-extraction technique named Gray Level Co-occurrence Matrix (GLCM) was utilized to classify breast lesions into normal and abnormal classes. The highest accuracy of 89.02% was achieved from the SVM classifier using a dataset of 330 mammograms. The AdaBoost feature selection technique was explored and as the image preprocessing step, only the cropping method was employed. Moreover, no augmentation technique is carried out in this study. Another research [10] suggested a novel strategy for accurately detecting breast lesions. Image-processing techniques were utilized to ready the mammography pictures for the feature and pattern extraction procedure in the first phase of this method. In the second phase, the collected features were used as inputs for two different supervised learning models, including the Backpropagation Neural Network (BPNN) and the LR models. A machine learning model for the BPNN was created, with the neural network model’s Logical Regression that achieved 93% accuracy. The authors [11], with the Wisconsin Diagnosis Breast Cancer dataset, used machine learning to determine the stages of breast cancer. Three widely used algorithms (RF, KNN and Naive Bayes) were compared in terms of breast cancer prediction. Among them all, the RF received the highest accuracy of 94%. An overview of the entire literature review including the previous techniques and their limitations is presented in Table 1.

## 4. Materials and Methods

**Figure 1:**
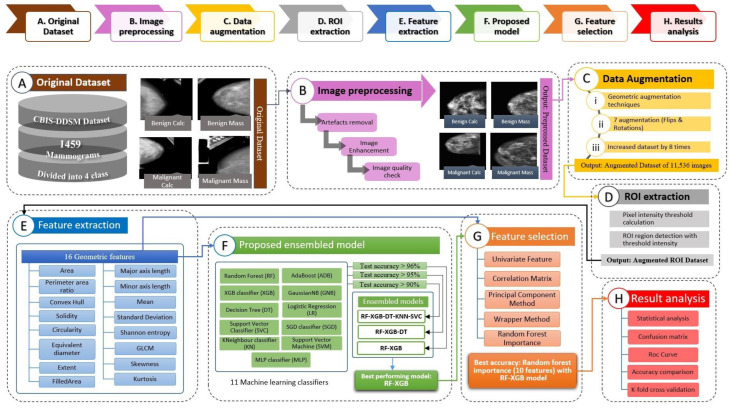
Complete process flow of this study. (**A**) Original CBIS-DDSM dataset. (**B**) Image-preprocessing steps including artefacts removal and image-enhancement methods. (**C**) Data-augmentation process with various geometrical features. (**D**) ROI extraction process from augmented images. (**E**) Feature extraction process with a total of 16 geometrical features. (**F**) Proposed Ensemble model generation by experimenting with various ML classifiers. (**G**) Experimentation with various feature selection methods using the suggested model. (**H**) Result analysis of the proposed approach.

The dataset utilized in this investigation is described in this section and all the methods of data preprocessing including artefact removal, image enhancement and ROI extraction. For artefact removal, methods such as Binary Masking, Largest Contour Detection, Canny Edge Detection and Hough Lines transformation method are performed. Afterwards, in the enhancement step, Gamma Correction and CLAHE are employed. The newly generated enhanced image dataset is augmented to increase the volume of the dataset. Afterwards, ROI is extracted from the augmented dataset using the region-growing algorithm with dynamic threshold values. Lastly, as described already, geometrical features are extracted from the augmented ROI dataset and an optimal model is developed with the highest performance. For boosting the performance even further, feature selection methods are employed. Figure 1 depicts the entire study procedure in detail.

### 4.1. Dataset

**Figure 2:**
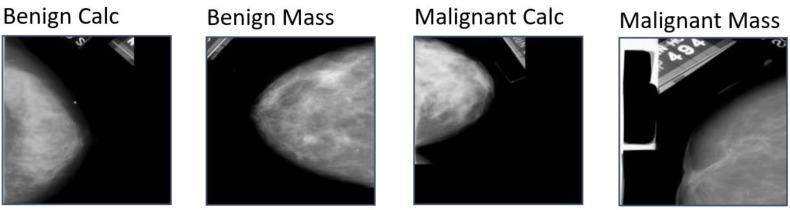
Images from each class of CBIS-DDSM dataset.

In this study, a total of 1459 mammograms are used which are collected from the CBIS-DDSM [12]. The dataset contains a total of four classes namely Benign calc (BC), Benign mass (BM), Malignant calc (MC) and Malignant mass (MM). Among them, 417 mammograms are found on BC, 398 images on BM, 300 images on MC and the rest of the 344 images on MM, which is shown in Figure 2. The sample of this dataset contains mammograms of the craniocaudal (CC) view which is considered as a standard view in mammogram screening. CC view ensures the most breast tissue in a mammogram without showing pectoral muscle as less as possible. Moreover, both breasts of a patient are not included in this dataset, only the breast of a particular patient showing signs of abnormalities are presented here. Moreover, All the mammograms are of dimensions 224 × 224 pixels and are in Red Green Blue (RGB) color format.

Description of the CBIS-DDSM dataset is provided in Table 2.

### 4.2. Challenges of the Mammography Dataset in Classification

To discriminate between benign and malignant lesions, particularly in mammograms, different structural changes are taken into account and analyzed. To determine whether a mammogram contains a malignant or benign lesion or no lesion at all follows a well-accepted standard such as the Breast Imaging-Reporting and Data System (BI-RADS). For instance, regarding the images containing lesions, their status is frequently confirmed by the pathological analysis of biopsies, or if benign and un-biopsied, their status is verified by a long-term follow-up. In a mammogram, the fatty tissue appears as gray, dense tissue as white and a tumor as white. Employing different algorithms, the ROIs can be extracted based on the intensity and pixel color value. However, sometimes even for radiologists, this task becomes challenging due to the interference of dense tissues. Mammograms are quite a challenging dataset as it contains ROI regions that are quite complex [13]. As the objective is to extract meaningful features from the cancerous region (ROI), segmenting the ROI from the mammograms is quite crucial. Successful execution of this task means addressing various challenges of mammogram images such as:

- Various artefacts (large texts and marks) are present within the mammograms resembling the pixel intensity of the ROI region that can interfere with the ROI extraction process.
- Malignant tumors are mostly found with an irregular shape as well as ambiguous and blurred edges that make it tricky to determine the boundaries of ROI.
- Along with the masses, the surrounding area of the lesion is important to preserve to ensure no loss of the cancerous region in the segmented images.
- Poor brightness and contrast level can be seen in some mammograms.
- Structural complexity of the breast portion of the mammogram having a white line attached to it.
- Patients with dense breasts are found with dense breast tissues showing pixel intensity near similar to the cancerous tissues.
- Limited number of mammogram images can be found in the chosen dataset.
- Visually intra-class dissimilarity and inter-class similarity between BC, BM, MC and MM.

### 4.3. Image Processing

**Figure 3:**
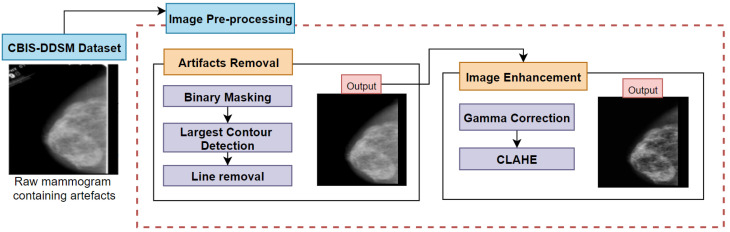
Illustration of entire image-processing techniques.

As the objective is to segment the cancerous region (ROI) from the mammograms, enhancing the mammograms can produce better segmentation of ROI regions. In this regard, various image-processing techniques can be a crucial step in enhancing the cancerous region of the mammogram [14]. Furthermore, irrelevant regions of the mammograms should be eliminated before the ROI segmentation phase as bright artefacts can interfere with the ROI extraction process. Therefore, there are mainly two processes in this section namely artefacts removal and image enhancement. Various steps utilized in this study for the preprocessing of the images are shown in Figure 3.

Firstly, artefacts from mammography are eliminated, using various algorithms (binary masking, largest contour detection [15]) in order to acquire a more precise ROI segmentation. Furthermore, to eliminate the presented vertical lines in the mammograms, Canny Edge Detection [16] and Hough Line transformation [17] algorithms are utilized. Secondly, to make the malignant lesion much more noticeable, image enhancement, a process of modifying the brightness and contrast of the original mammograms is used. Subprocesses involved in this stage include gamma correction [18,19] and CLAHE [20]. After employing CLAHE, visibility has been shown to improve. In order to test the quality of the mammograms, assessment methods such as MSE, RMSE, SSIM and PSNR are applied to the processed pictures in the verification step.

#### 4.3.1. Artefact Removal

**Figure 4:**
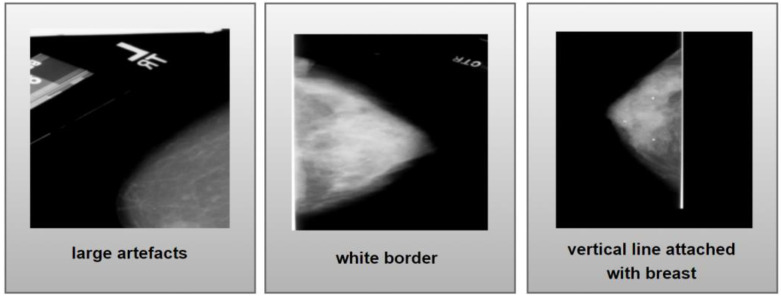
Various artefacts present in mammograms.

Various artefacts can be found on breast mammogram images that are shown in Figure 4 that can disrupt the segmentation process. Texts along with some large objects can be seen on the mammograms along with white bright lines that are attached in the breast area and the border of the images can be observed (Figure 4). These artefacts show a similar color intensity as the ROI regions, which can hamper the segmentation process later on. In this regard, the removal of these artefacts is quite crucial in the successful segmentation of ROI areas. With this view, the various artefact removal processes are utilized and described in this section.

##### Binary Masking

**Figure 5:**
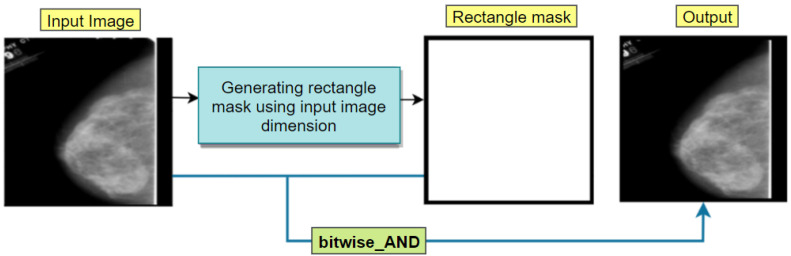
Removal of the white border with binary masking using a rectangle mask.

For any image in computer vision, every pixel is addressed by either ones or zeros. Python allows us to manipulate the bits and conclude which pixels to extract and which to eliminate. There are some border white lines that can be eliminated using the binary-masking method. The binary mask has 2 bits, of which ‘1′ stands for white and ‘0′ for black. In our experiment, cv2.rectangle() method of OpenCV python has been used which demands five parameters named border_color, input_image, border_thickness, end_point and start_point to make a mask of a rectangular shape with the exact size as our input image (height and width of 224 pixels and thickness of 5 pixels). Afterwards, the rectangular mask and the input images are combined and a border-free output image is achieved (Figure 5).

##### Largest Contour Detection

**Figure 6:**
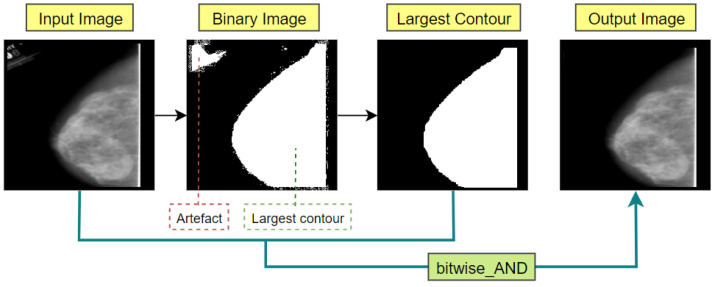
Largest contour detection to extract breast part of the mammogram.

Since the breast contour is the greatest contour shown on mammograms, artifact-free mammograms can be produced by extracting the largest contour. By using contour detection, the boundaries of each presented object of an image can easily be located. Using OpenCV, contours can be detected and mark the region with the function of *findContours()* and *drawContours()*. Figure 6 illustrates the complete process flow of extracting the largest contour.

Firstly, we read the mammogram images in grayscale format and converted those into binary format (Binary image in Figure 6). In this process, every object in the images that has a similar intensity value will be changed over to white (1). The image’s remaining pixels will be changed over to black (0). Every white pixel that is isolated by a black pixel will be taken into account as a contour. The next step was to locate all the contours in the mammogram images using the *findContours()* method. It produces a list containing all contours, where the max () function is utilized to quickly identify the largest one based on the contour areas. After getting the largest contour, the area above it is drawn using *drawContours()*, and a binary mask is returned that only contains the biggest blob. Afterwards, this binary mask is combined with the original image using bitwise_AND() function, obtaining an artefact-free image containing simply the mammogram’s breast section (output image of Figure 6).

##### Line Removal

**Figure 7:**
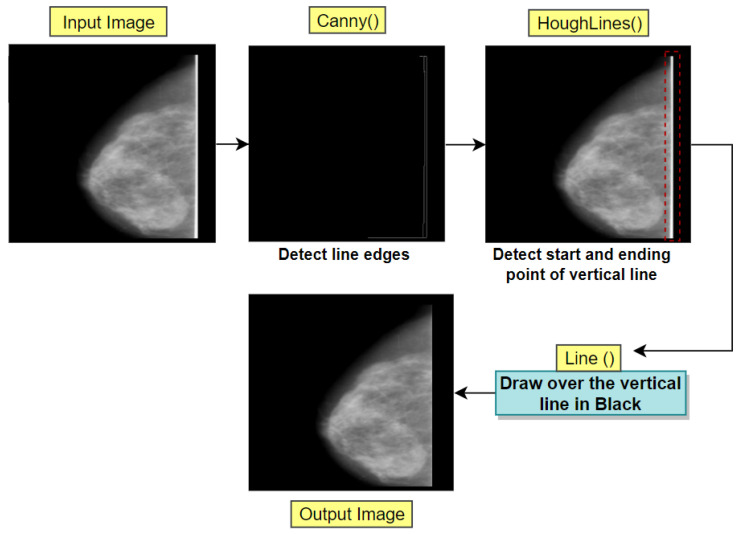
Removal of vertical white lines attached to breast.

While observing the resultant output image of the previous process (Figure 6), a vertical white line can be seen which is attached to the breast portion of the mammogram. This can be detected by marking the edges present in the image utilizing Canny Edge Detection and finding the start and end points of the vertical edge (white vertical line) using Hough Lines Transformation process of OpenCV. Afterwards, using OpenCV line drawing algorithm, a line matching the background color of the image (black) can be drawn over the detected vertical edge, thus removing it. Figure 7 illustrates the whole process of vertical line removal.

#### 4.3.2. Image Enhancement

**Figure 8:**
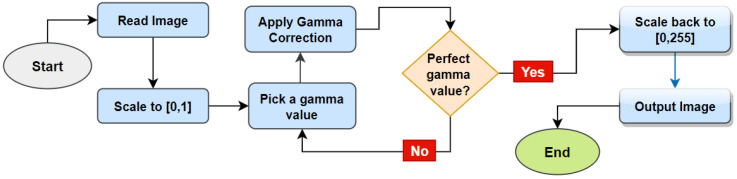
Gamma correction process.

In this segment of our research, we applied gamma correction and the CLAHE method so that the pixels’ contrast and brightness can be better adjusted. Firstly, Equation (1) is used to adjust the contrast and brightness of the photographs using the Gamma Correction approach:

(1) $$V_{\mathrm{out}\;}=AV_{\mathrm{in}\;}^{\gamma }$$

here $V_{\mathrm{in}\;}$ denotes the positive real input value and it is raised to the power γ (gamma). Afterwards $V_{\mathrm{in}\;}^{\gamma }$ is multiplied with constant A for having the value $V_{\mathrm{out}}$. The value of γ controls the overall image brightness and contrast levels. For a gamma value < 1 image will be darker, any value of > 1 results in the image brighter and a value of 1 for gamma has no effect. After applying the gamma value, if the image becomes too dark or too bright, this value can be altered and tested with the image until the best output is gained. In this process, a suitable gamma value is chosen and gamma correction is employed. The complete gamma correction process is shown in Figure 8.

**Figure 9:**
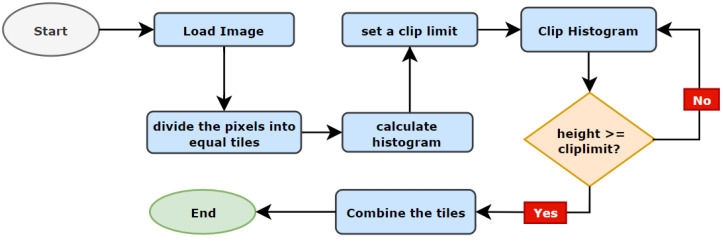
CLAHE process.

The CLAHE, which is a version of Adaptive Histogram Equalization (AHE), is utilized to improve visibility when contrast over-amplification occurs in poorly contrasted images. CLAHE emphasizes tiles, which are little portions of an image rather than the entire picture. The close-by tiles are combined using bilinear interpolation to get rid of the arbitrary borders. Figure 9 illustrates the complete process of CLAHE.

**Figure 10:**
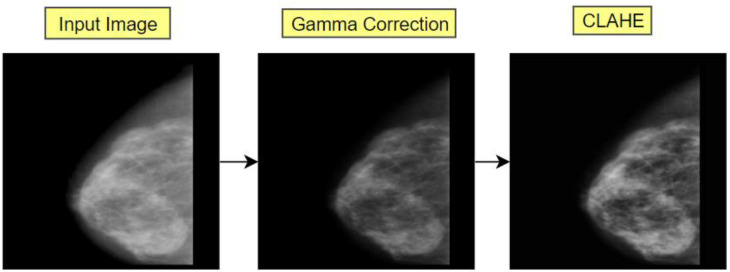
The transformation of artefact removed image after applying gamma correction and CLAHE.

There are two parameters to utilize while using CLAHE: Clip Limit and TileGridSize. The Clip Limit parameter is used for determining the contrast limiting threshold whereas the value of TileGridSize specifies the number of tiles in each row and column. In this process, an image is partitioned into little equivalent measured lumps called ‘tiles’. For each tile, a histogram is calculated and a clip limit is set to disseminate the contrast in a balanced manner. The histogram is clipped in such a way that its height is below the clip limit. At last, those partitioned pieces are consolidated again with bilinear interjection. The resultant image of gamma correction and the CLAHE process is presented in Figure 10.

Selected parameter values of all algorithms regarding artefact removal and image enhancement process are presented in Table 3 for better understanding.

#### 4.3.3. Assurance of Image Quality

Some statistical analyses have been performed to make certain that the image quality has not been compromised even after applying several image processing algorithms. Firstly, a total of ten artefact-removed images denoted as AF_images are randomly selected. Secondly, gamma correction and CLAHE are applied to these ten images, which are denoted as E_images. Afterwards, comparing these ten enhanced images (E_images) with artefact-removed images (AF_images), the value of root mean squared error (*RMSE*), structural similarity index measure (SSIM), peak signal-to-noise ratio (*PSNR*) and mean squared error (*MSE*) [21] are calculated to make certain that the image quality does not degrade and the image information is well preserved in the enhanced images (Table 4).

*MSE* characterizes the combined squared error among pixels contained in the two images. Worth more than 0.5 means the quality has been diminished. Worth = 0 presents that image is totally commotion free and of optimum quality. *MSE* can be calculated using Equation (2).

(2) $$MSE=\frac{1}{pq}\sum_{i=0}^{m-1}\sum_{i=0}^{n-1}{\left(O\left(m,n\right)-P\left(m,n\right)\right)}^{2}$$

where the ground truth image is denoted by O; images after processing are denoted by *P*; pixels of O and *P* are denoted by *p* and *q*. Lastly, the rows and columns of pixels are denoted by *m* and *n*.

*PSNR* signifies the ratio between a sign’s most extreme conceivable power and also the power of the corrupting noise impacting the image quality which is calculated with Equation (3).

(3) $$PSNR=20\log_{10}\left(\frac{\left(MA\bar{X}\right)}{\sqrt{MSE}}\right)$$

here maximum pixel values contained in the image is denoted by $MA\bar{X}$. Regarding the 8-bit image, an acceptable PSNR value is usually between 30 and 50 decibels [21].

SSIM (Structural Similarity Index) measures the decrease in image quality brought about by preprocessing algorithms and this can be derived using Equation (4).

(4) $$\mathrm{SSIM}\left(x,y\right)=\frac{\left(2\mu_{x}\mu_{y}+c_{1}\right)\left(2\sigma_{xy}+c_{2}\right)}{\left(\mu_{x}^{2}+\mu_{x}^{2}+c_{1}\right)\left(\sigma_{x}^{2}+\sigma_{y}^{2}+c_{2}\right)}\;$$

*RMSE* is a measurement that quantifies the difference in image quality between original and enhanced mammograms. A lower *RMSE* value (close to zero) indicates lesser errors and ensures better image quality. The *RMSE* value is calculated with Equation (5).

(5) $$RMSE={\left[\sum_{j=1}^{N}{\left(d_{f_{i}}-d_{d}\right)}^{2}/N\right]}^{\frac{1}{2}}\;$$

here $d_{f_{i}}$ is the artefact-removed image and $d_{d}$ denotes the enhanced images. The squared difference is denoted with ${\left(d_{f_{i}}-d_{d}\right)}^{2}$ and dataset size is indicated by N.

Calculated statistical values are presented in Table 4 which contains PSNR, *MSE*, *RMSE* and SSIM values between randomly selected 10 images from the artefact-removed mammogram and enhanced mammograms of these 10 images.

It is evident from Table 4 that the quality of the images is well preserved and no loss of information is observed in the processed images.

### 4.4. Data Augmentation

**Figure 11:**
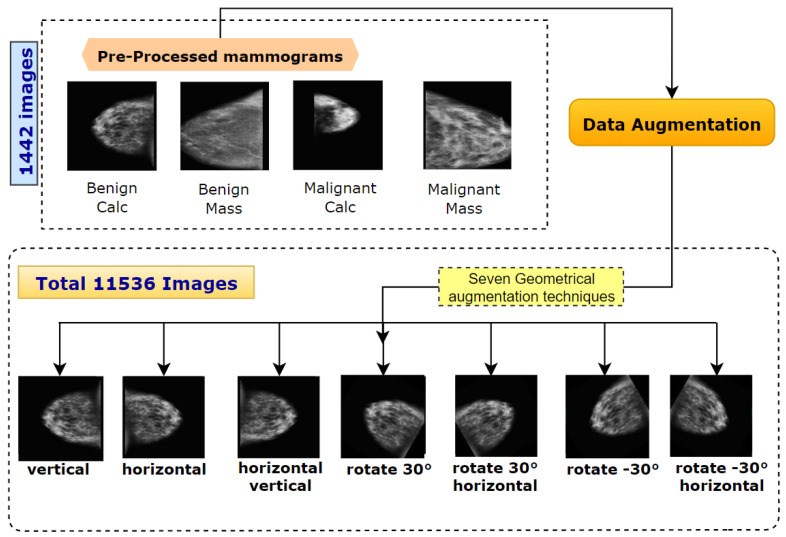
A total of seven geometric augmentation methods applied in processes dataset.

Data augmentation is crucial in developing the performance and outputs of ML algorithms by producing new and diverse samples of the input dataset. A total of seven different augmentation methods (Figure 11) on the preprocessed dataset have been employed [14]: (1) flipping horizontally, (2) flipping both horizontally and vertically, (3) flipping vertically, (4) 30° rotation, (5) 30° rotation and flipping horizontally, (6) −30° rotation and (7) rotating −30° and horizontal flip. Therefore, by increasing the original dataset eight times (including the original ROI images), a dataset of 11,536 mammograms is created.

### 4.5. ROI Extraction

The most important medical imaging procedure is image segmentation, which isolates the region of interest (ROI) and makes it easier to extract various properties of the ROI [22]. Along with the meaningful and necessary regions, an image can contain irrelevant regions that might reduce the expected model performance. Therefore, the successful extraction of meaningful pixels (ROI) can have an impact on accelerating the overall processing performance, especially while working with ML models. ROI extraction can be achieved by utilizing the region-growing algorithm with a dynamic intensity threshold value depending on every image.

**Figure 12:**
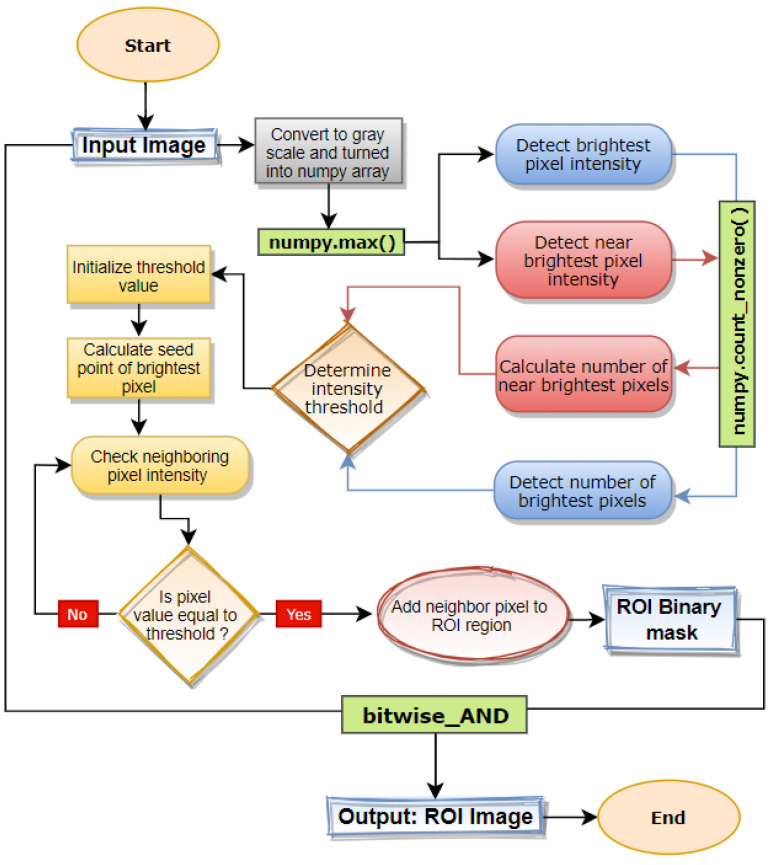
Illustration of entire ROI segmentation process.

In Mammograms, the cancer region or ROI is represented as a bright spot with a higher intensity level than the rest of the mammogram [23]. In this regard, the breast part that appears brighter on a mammogram is potentially more crucial in the successful detection of breast cancer [24]. In some cases, dense breast tissue can be observed in mammograms, which also appear brighter with a high-intensity level [25] that is slightly lower than the intensity of ROI. In this regard, a universal intensity threshold value does not suffice in the successful extraction of ROI for every image. To solve this issue, a dynamic approach is taken for calculating the highest intensity threshold value for every image. The entire segmentation approach is picturized in Figure 12.

In this research, instead of applying fixed intensity threshold values, a dynamic procedure is performed where the brightest pixel value of each mammogram along with the number of the brightest pixels and near brightest pixel is computed making this a dynamic intensity threshold approach ROI extraction.

Firstly, preprocessed mammograms are taken as the input and converted from RGB to grayscale. Afterwards, using the *max()* function, the highest pixel intensity is calculated. While segmenting only with the highest intensity, some ROI neighboring regions containing near the highest pixel intensity might be eliminated. To avoid this problem, the near highest pixel intensity is also calculated. Afterwards, the number of brightest pixels and near brightest pixels are counted using *count_nonzero(*) function. In most cases, the near-bright pixel count (bp) happens to be the greater than brightest pixel count (nbp). After extensive experimentation with the processed mammogram images, some conditions are derived based on the pixel count of both bp and nbp. With the experimented conditions, the intensity threshold is determined for that particular image.

**Figure 13:**
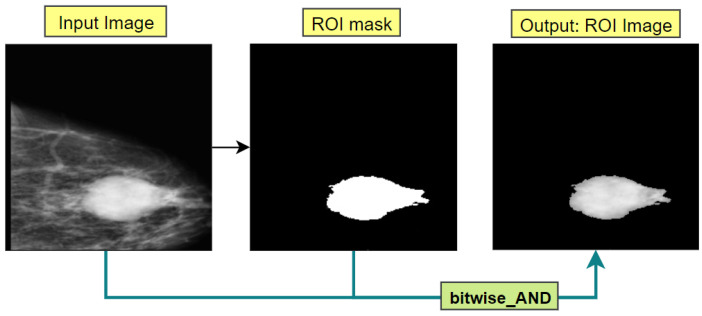
ROI extraction process.

Afterwards, the middle point of the brightest pixel region is calculated and this middle point is determined as the seed point. Region growing algorithm takes the seed point and determines the intensity threshold value accepting the image as input. The algorithm starts from the seed point pixel and examines all neighboring pixels. If a neighboring pixel shows an intensity level higher or equivalent to the inputted intensity thresholding value, the neighboring pixel is added to the ROI region. The algorithm stops upon finding no similar-intensity pixels in the image. The algorithm then returns a binary mask of the calculated ROI where the ROI is white and the rest of the image is black. Finally, utilizing the *bitwise_AND()* function of open CV, the binary mask is merged with the input image and regions of the mask image containing white pixels are extracted from the input image; thus, we can achieve an image containing only the ROI area. The output of this process is shown in Figure 13. The whole process is conducted for each image resulting in different intensity threshold values depending on the individual intensity level which contributes to better ROI segmentation.

## 5. Proposed Approach

As discussed, our proposed ensemble model is developed after a couple of experiments with eleven ML algorithms and 16 geometric features. In this part, numerous features are derived from the segmented ROI pictures that have undergone preprocessing [26] and eleven ML models are used for classification into four classes: BC, BM, MC and MM using geometrical features of ROI images. At the end of the section, the optimal model configuration which is developed by the stacking method and different feature selection techniques are described.

### 5.1. Machine Learning Algorithms

A total of eleven cutting-edge Machine learning (ML) algorithms: Decision Tree (DT), AdaBoost (AB), Logistic Regression (LR), Random Forest (RF), XG Boost (XGB), K Nearest Neighbors (KNN), Support Vector Classification (SVC), Multilayer Perceptron (MLP), Support Vector Machine (SVM), Gaussian Naive Bayes (GNB) and Stochastic Gradient Descent (SGD) have been used in this study for classifying breast cancer using the mammogram extracted features.

### 5.2. Feature Extraction

A variety of geometric features were extracted from ROI images of the augmented dataset and a numerical dataset was created for classifying the mammograms with machine learning algorithms. A total of 16 geometrical features [27] of the ROI were extracted as presented in Table 5 that are widely used for image classification.

**Figure 14:**
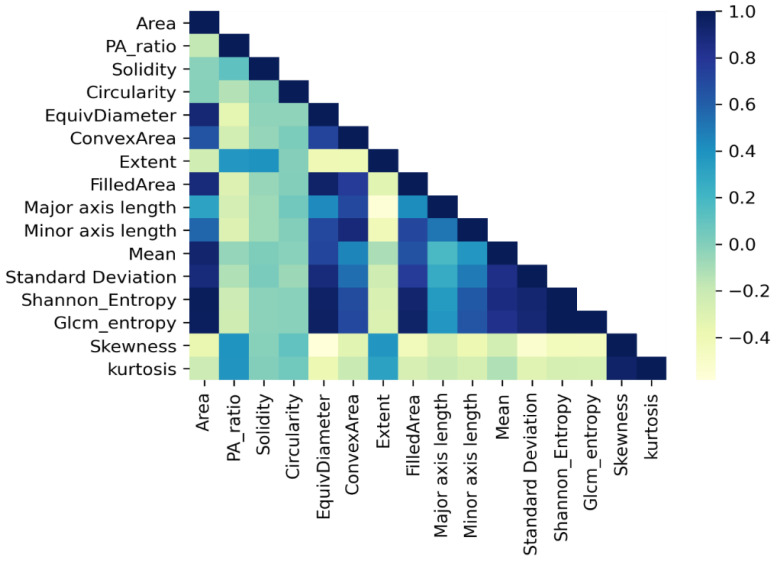
Correlation between extracted features.

The feature correlation matrix is illustrated in Figure 14 which shows the characteristics of the features. It is evident that features Filled area, Minor axis length, Major axis length, mean Standard deviation, Shannon entropy and GLCM entropy show a strong correlation with Equivalent diameter, Convex area and area. Furthermore, a noticeable correlation between Kurtosis and Skewness can be observed. On the other hand, a considerably weak correlation can be observed for Skewness and Equivalent diameter, Major axis length and Extent. It can be observed that the Pa ratio and Extent have the lowest correlation with other features. The importance of these features can be concluded after experimenting with various ML models and observing the results of various feature selection techniques.

### 5.3. Training ML Algorithms

**Figure 15:**
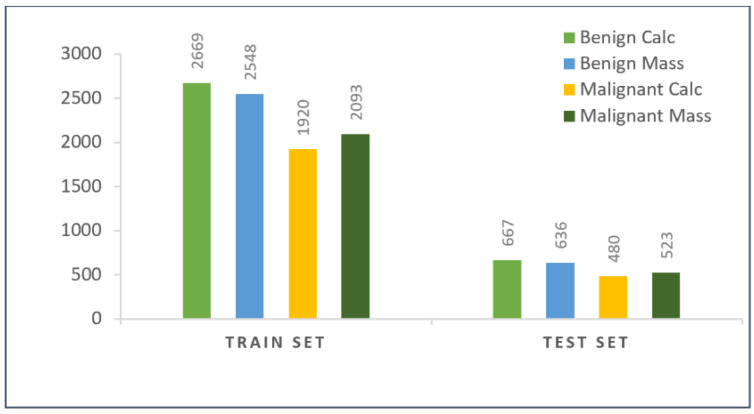
Class distribution of test and train set after splitting the augmented dataset.

The whole numerical dataset of extracted features was split into a test set and a train set, randomly maintaining 80:20 ratios, respectively. The train set contains features of 9230 mammograms and the test set contains the features of 2306 mammograms. It is displayed by how the mammograms were distributed among all four classes of the training set and testing set in Figure 15.

The class labels were converted from string to numerical values (BC to 0, BM to 1, MC to 2 and MM to 3). Furthermore, all the features are normalized by rescaling the numeric values within a range of 0–1. For training and validating all ML models, K-fold cross-validation has been used with a K value of 10 [39]. Afterwards, for evaluating the models, the prediction of the test set was performed acquiring relevant information on the performance of the models on unseen test data. Furthermore, other evaluation matrices are also calculated for better evaluation of the ML models on the numerical dataset.

### 5.4. Proposed Ensemble Model: RF-XGB-10

Depending on the 11 ML model’s performance, three ensemble models are generated by stacking where the Random Forest–XGB classifier is found to have performed best with the highest accuracy. The model is titled RF-XGB-10 as, after feature selection, we get 10 optimal features for which the highest performance is obtained.

Random Forest depends on the beneficial aspects of a random vector inspected freely and comparable dispersion throughout all the forest’s trees. [40]. The generalization error constricts to a certain size as a forest’s tree number increases. The generalization error of a forest of tree classifiers is influenced by the strength of the individual trees within the forest and their relationship. Random forests are built by aggregating the N Number of decision trees where the tree prediction values are the average of all individual predictions of trees [41].

(6) $$A_{k,\mathrm{pred}}=\frac{1}{T}\sum_{t=1}^{T}A_{k,t,\mathrm{pred}}\;$$

here the prediction value of the activity of the *k*-th compound by RF is denoted by $A_{k,t,\mathrm{pred}}$. The total amount of trees is denoted by T and the prediction value of the activity of the *k*-th compound by *k*-th tree is denoted by $A_{k,\mathrm{pred}}$.

Extreme Gradient Boosting (XGBoost) is a state-of-the-art algorithm which is an end-to-end, scalable tree-boosting system. [42]. To reduce overfitting, the final learnt weights are smoothed using the regularized learning approach. Models that use fundamental, predictive functions will be preferred by the regularized objective. Two more strategies are employed in addition to the regularized objective to prevent overfitting. First, shrinkage, which scales freshly added weights after each round of tree boosting by a certain amount and secondly, column (feature) subsampling which prevents over-fitting more than the traditionally used row sub-sampling methods. Utilization of column sub-samples can speed up computations of the parallel algorithm. These features make XGBoost faster than other cutting-edge algorithms and it dominates structured datasets in terms of classification problems. Combining RF and XGB ML models using the staking method, the output of these two models runs through the default meta learner (Logistic Regression) that combines the learned weights of both models while minimizing the weakness of RF and XGB and maximizes the strength of these two models and decreases error rate.

## 6. Results and Discussion

This section explores the effectiveness of 11 machine learning models to develop an optimal ensemble model for this classification problem. Furthermore, various feature selection techniques have also been applied to improve the performance of the developed model even further.

### 6.1. Evaluation Matrices

Various statistical measures including Accuracy (ACC) [43], Matthews Correlation Coefficient (MCC), F1 score [43] and AUC value are calculated for evaluating the performance of the machine learning models [44]. All of these values can be calculated with True positive (TP), False positive (FP), True negative (TN) and False negative (FN) values which can be achieved from the confusion matrix.

(7) $$\mathrm{ACC}\;=\frac{\mathrm{TP}+\mathrm{TN}}{\mathrm{TP}+\mathrm{TN}+\mathrm{FP}+\mathrm{FN}}\;\left(6\right)$$

(8) $$\mathrm{MCC}=\frac{\mathrm{TP}\cdot \mathrm{TN}-\mathrm{FP}\cdot \mathrm{FN}}{\sqrt{\left(\mathrm{TP}+\mathrm{FP}\right)\cdot \left(\mathrm{TP}+\mathrm{FN}\right)\cdot \left(\mathrm{TN}+\mathrm{FP}\right)\cdot \left(\mathrm{TN}+\mathrm{FN}\right)}}\;\left(7\right)$$

(9) $$F_{1}\;\mathrm{score}\;=\frac{2\cdot \mathrm{TP}}{2\cdot \mathrm{TP}+\mathrm{FP}+\mathrm{FN}}\;$$

### 6.2. Comparison of Different ML Models Based on Accuracy Measures

Table 6 showcases the performance of 11 hyper-tuned machine learning models that were applied to the dataset. It was observed that among the 11 models, RF achieved the best performance with 95.91% test accuracy followed by XGBoostClassifier with 95.40% accuracy. Furthermore, the DecisionTree classifier achieved a test accuracy of 94.62% and both KNeighbors and Support Vector classifier performed moderately with 92.82% test accuracy.

### 6.3. Developing Optimal Ensembled Model

**Figure 16:**
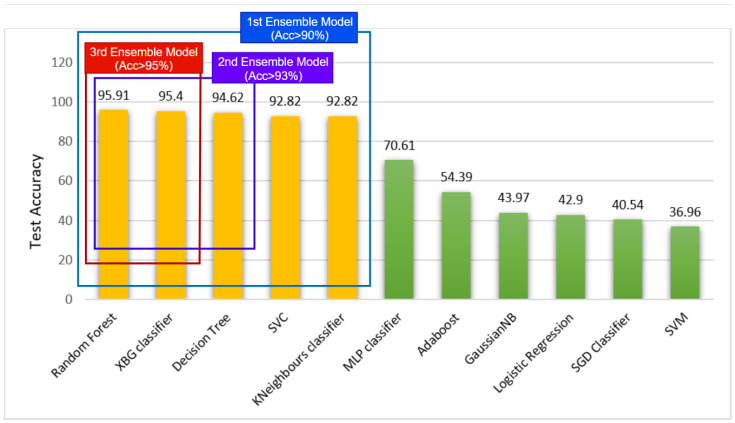
Ensemble model generation approach.

In order to develop the optimal ensemble model, a stacking method was employed to combine multiple ML models to formulate the desired model. It is visible in Table 6 that five models among the 11 models perform with the highest accuracies above 90%. A total of three stacked models were developed based on these five ML models (Table 7). Figure 16 illustrates the generation process of the three ensemble models.

Firstly, models with accuracies above 90% were considered to make the first ensembled model RF-XGB-DT-KNN-SVC by stacking RandomForest, XGBoost, DecisionTree, KNearestNeighbors and SupportVector classifiers and trained the model within our numerical dataset. It can be seen from Table 7 that this model performed with a test accuracy of 91.53%. Furthermore, we generate the second ensemble model by stacking ML models with accuracies above 93%, RandomForest, XGBoost and DecisionTree classifier creating the RF-XGB-DT model. It is observed that this model records a test accuracy of 95.64% with our dataset. Finally, models with accuracies above 95% were stacked together and RF-XGB is developed using RandomForest, and the XGBoost classifier is developed which yields the highest performance. Performances of these three models are showcased in Table 7. It is evident that the RF-XGB classifier gained a test accuracy of 96.57% which is a 0.66% accuracy gain over the previously obtained test accuracy for RF demonstrated in (Table 6).

### 6.4. Feature Selection

**Figure 17:**
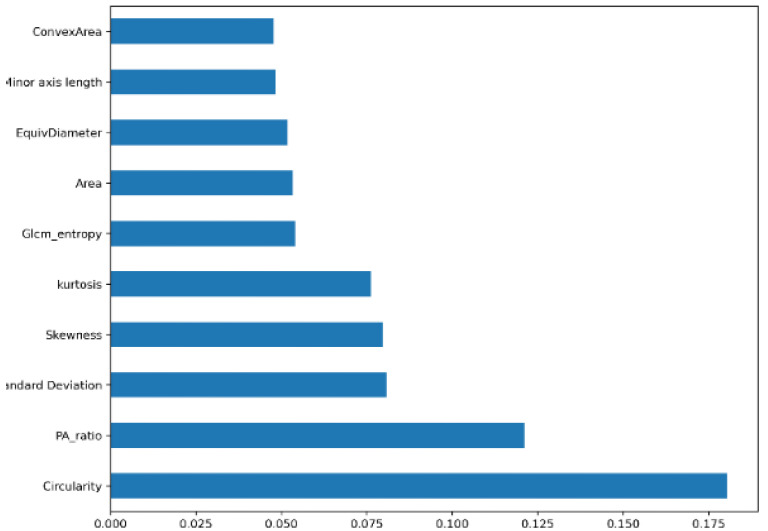
Random Forest importance feature selection.

For feature selection, some well-known algorithms: Random Forest feature importance (RF) [45], Univariate features, Correlation Matrix [46], Principal Component Analysis method (PCA) [47] and Wrapper Method [48] with various configurations are used in this study. Table 8 contains the performance analysis of the RF-XGB model trained on the selected features of various feature selection techniques. The Random Forest importance algorithm, with a threshold of 0.045 (Figure 17), resulted in 10 selected features that outperformed all other techniques (Table 8) with a test accuracy of 98.05% and an AUC value of 98.91%.

It can be observed from Table 8, after conducting various feature-selection methods, that the test accuracy did not decrease drastically for any test case and remained above 96%. This further justifies that the extracted features are quite effective and most of the features play a significant role in the successful classification of breast cancer into four classes.

### 6.5. Performance Evaluation

**Figure 18:**
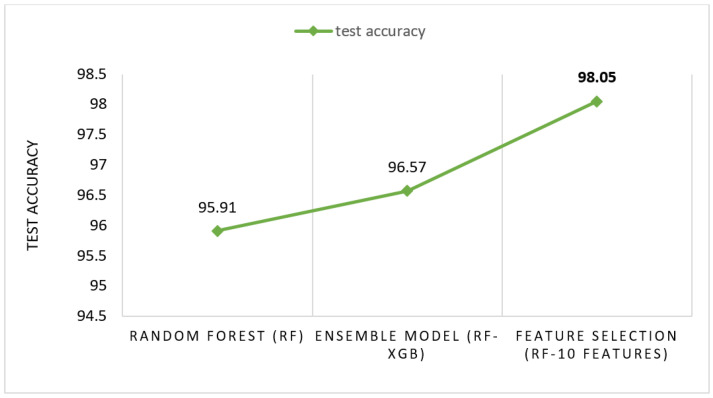
Gradual increase in test accuracy.

**Figure 19:**
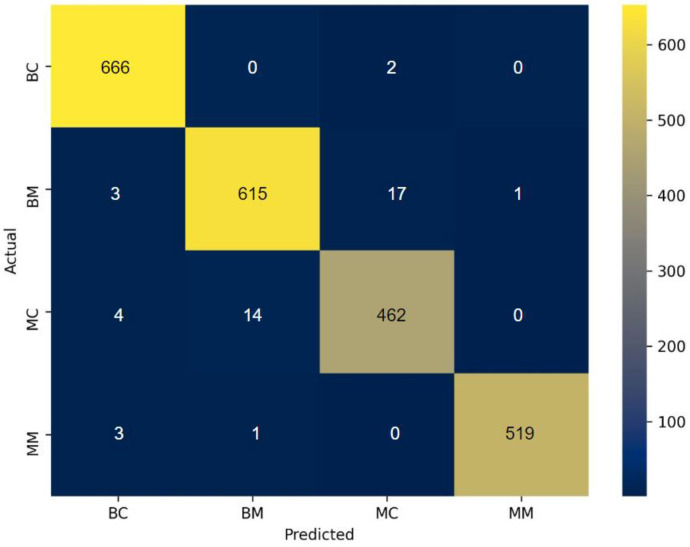
Confusion matrix of RF-XGB model.

**Figure 20:**
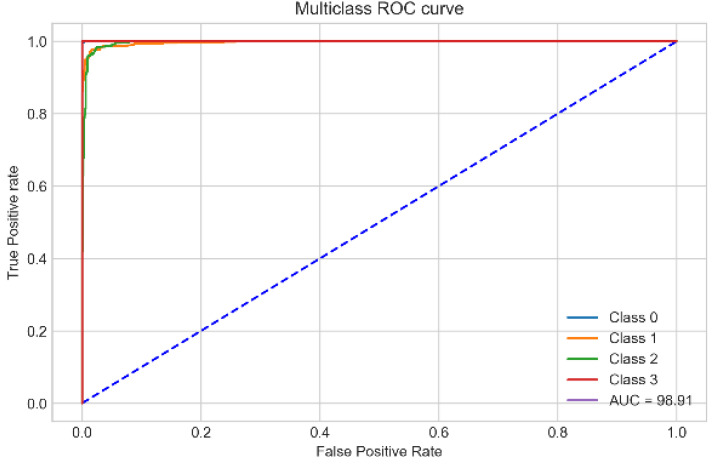
ROC curve of RF-XGB-10 model.

The proposed RF-XGB-10 model outperforms other approaches by achieving a test accuracy of 98.05%, MCC of 97.27%, F1-Score of 98.05% and AUC of 98.91%. The performance of the proposed model increased gradually as various methods are introduced. For a better understanding, a visual representation is given in Figure 18. Moreover, for the evaluation of the model, Figure 19 and Figure 20 demonstrate the confusion matrix and ROC curve, respectively, for the RF-XGB model trained and tested on 10 selected features with a feature importance algorithm named Random Forest.

It is evident from the confusion matrix Figure 19 that the RF-XGB classifier can produce higher true positive predictions across all classes and a very low number of false predictions were observed in all classes. Overall, this proves that the model is not prejudiced toward any individual class and is capable of predicting all the classes with reasonable consistency.

It is observed from the ROC (Figure 19) that the curves of all four classes nearly meet at the top left corner, indicating a highly correct prediction across all classes with close to no false predictions. This supports the efficacy of the suggested strategy even more, with a high AUC value of 98.91%.

**Figure 21:**
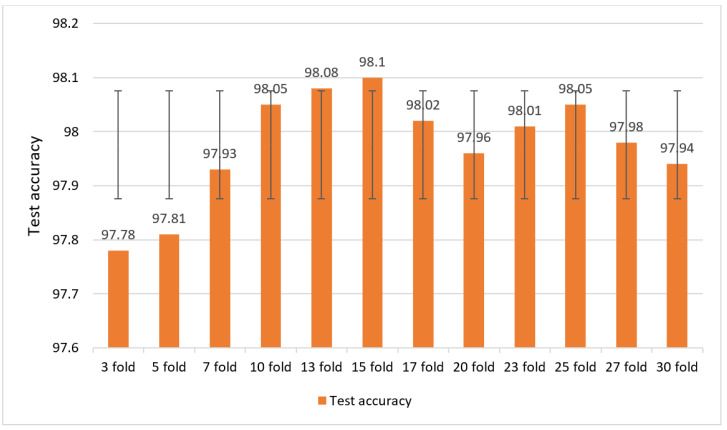
Results of K-fold cross-validation test with different K values ranging from 3 to 30.

Our proposed RF-XGB-10 model performs with optimal accuracy (>97%) across all the feature selection techniques of fourteen cases which validates the performance consistency of the model. In order to evaluate the performance consistency even further, we put the model to the test using twelve cross-validation configurations with different K values ranging from 3 to 30. Each K-fold cross-validation’s results are displayed in Figure 21.

This observed that the model is able to produce a good performance for all K-folds. (>97.7%). None of the folds saw a significant deterioration in performance, which validates our model’s robustness even more. It also symbolizes superiority in the feature extraction process, as well as THE Random Forest importance feature selection method for which the proposed model can produce a performance that is consistent with a gradual increase in the test accuracy.

### 6.6. Comparison with Some Existing Literature

In this segment, the proposed approach for classifying breast cancer using mammograms is further evaluated by comparing its performance against some recent studies. This comparison is made in terms of test accuracies of recent works which are showcased in Table 9. The SVM classifier is utilized by Vijayarajeswari et al. [7] for the classification of breast cancer into three classes with a test accuracy of 94%. Similarly, Meselhy Eltoukhy et al. [8] achieved 95.84% accuracy by utilizing the SMV classifier and wavelet coefficient method for feature extraction. A different approach is taken by Tang et al. [6] utilizing the voting classification method with a test accuracy of 96.06%. All the papers discussed in this comparison implemented two or three class-based classifications and no data augmentation technique is employed. In our proposed approach, we have used a dataset of 1459 mammograms which is later increased to 11,1536 mammograms by augmentation. The approach can beat the performance of all the studies shown in Table 9 by achieving the highest accuracy of 98.05%.

### 6.7. Discussion

This study’s key contribution is to identify a robust ML algorithm with the highest possible accuracy gained by performing extensive image processing, segmentation and effective feature selection methods. While training an ML algorithm with geometric features, the features should be as relevant as possible to achieve optimal performance. Without segmenting the images properly, unnecessary regions would have existed on the images. Therefore, only the necessary and meaningful ROI is used in this research to extract the features as the presence of artefacts, noise and surrounding breast tissues might compromise the accuracy achieved. Moreover, while extracting the ROI, for a particular threshold value for every image, necessary information may not be extracted properly. Hence, a dynamic ROI extraction procedure has been introduced. Furthermore, data augmentation is another crucial technique that aids in improving accuracy. The optimal ML algorithm is determined and developed by means of thorough experimentation with the feature dataset. In this regard, the ensemble method has proven to be an efficient way of getting high accuracy. However, in our study, instead of randomly picking ML algorithms to stack, we have determined the algorithms based on the accuracy gained on our dataset. Finally, a number of feature selection methods were incorporated which resulted in notable performance improvement. According to the findings of this study, optimal performance, even on a complex small dataset having artefacts, can be gained by means of suitable image preprocessing and feature selection and model-building techniques.

## 7. Conclusions

This study proposes a geometrical feature-extraction method for extracting features from mammogram images. After preprocessing and extracting ROI from the images, 16 geometrical features were extracted from the ROIs. Eleven popular ML algorithms have been applied using the geometrical features to find the optimal models in terms of the highest accuracy. Three ensemble models are generated by stacking five top-performing models following the threshold of test accuracies of >90%, >93% and >95%. Among the three, the Random Forest–XGBoost ensemble model outperformed other well-performing models. Experimentations with various feature selection techniques were employed to further enhance the performance of the ensemble model resulting in our proposed model RF-XGB-10 with a test accuracy of 98.05%. Moreover, several image-preprocessing techniques and the introduction of the dynamic segmentation approach aid in segmenting the ROI effectively, which results in improving overall performance. The approach proposed in this study can accurately classify several abnormalities in breast tissues which will be significantly useful in practical applications especially for clinicians.

## Figures and Tables

**Table 1:** An overview of the literature review, including past methodologies and limitations.

Authors	Task	Models	Limitations
Tang et al. [6]	Classification	Backpropagation Network, Naïve Bayes Classifier and Linear Discriminant Analysis	i. Lack of image enhancement techniques. ii. Artefact removal is not conducted iii. Absence of data-augmentation technique
Vijayarajeswari et al. [7]	Classification	SMV	i. Absence of data-augmentation technique ii. Experimentation with various models is missing
Meselhy Eltoukhy et al. [8]	Classification	SMV	i. Lack of image-enhancement techniques. ii. Artefact removal is not conducted iii. Absence of data-augmentation technique iv. Experimentation with various ML models is absent
Singh et al. [9]	Classification	Random forest	i. Lack of automatic ROI segmentation process ii. Absence of data-augmentation techniques
Al-Hadidi et al. [10]	Segmentation Classification	Logistic Regression and Backpropagation Neural Network	i. Lack of automatic ROI segmentation process ii. Absence of data-augmentation techniques iii. Experimentation with various ML models is absent

**Table 2:** Narration of CBIS-DDSM dataset.

Samples in dataset (total)	1459 mammograms
Dimension	224 × 224 pixels
Color Grading	Red Green Blue (RGB)
Benign Calcification (BC)	398 mammograms
Benign Mass (BM)	417 mammograms
Malignant Calcification (MC)	300 mammograms
Malignant Mass (MM)	344 mammograms

**Table 3:** Methods used for artefact removal and image enhancement.

Algorithms	Functions	Values of Parameter
Binary masking	OpenCV rectangle()	Width = 5
Largest contour detection	OpenCV *findContours()*	Mode for contour approximation = CHAIN_APPROX_SIMPLE Retrieval mode of contour = RETR_EXTERNAL
	*max()*	Meassure key = contourArea
	OpenCV *drawContours()*	Index = largest contour, color of contour boarder = (255, 255, 255), width = 1
Vertical line removal	OpenCV Canny()	Minimum Value = 50, maximum Value = 150 and Size of aparture = 3
	OpenCV HoughLines()	edges = Canny(), rho = 1, theta = numpy. pi/50, threshold = 50
	Line	Color value = (0,0,0), Width = 5
Gamma correction	Numpy array()	Value of gamma = 2.0
CLAHE	OspenCV createCLAHE()	Clip Limit = 1.0, tile Grid Size = (8, 8)

**Table 4:** PSNR, MSE, RMSE and SSIM scores for random ten images of the dataset.

Image	PSNR	MSE	RMSE	SSIM
Img_1	36.67	16.38	4.04	0.958
Img_2	36.29	14.73	4.21	0.959
Img_3	37.28	15.35	3.91	0.965
Img_4	38.31	14.41	3.79	0.961
Img_5	39.67	12.63	3.55	0.974
Img_6	40.29	12.35	3.51	0.974
Img_7	38.28	14.69	3.83	0.966
Img_8	40.16	13.39	3.65	0.968
Img_9	36.84	14.32	3.78	0.964
Img_10	39.17	15.42	3.92	0.969

**Table 5:** Extracted geometrical features from ROI images.

No	Feature Name	Feature Definition
1	Area [28]	The total area of all extracted regions
2	Perimeter area ratio [28]	The ratio between the measure of the length of a shape around the ROI and Area
3	Convex Hull [29]	The set of pixels that are included in convex polygon that is smallest surrounding white pixels
4	Solidity [30]	Contrasting object areas compared to its Convex Hull by utilizing the pixels that make up the Convex Hull.
5	Circularity [31]	The measurement of the roundness of the ROI
6	Equivalent diameter [32]	This is the diameter of a circle that has the same perimeter as the ROI region.
7	Extent	The area of the ROI divided by the Area of Convex hull
8	FilledArea	The total area measurement of only the ROI regions
9	Major axis length [27]	The longest length of the ROI object
10	Minor axis length [27]	The smallest width of the ROI object
11	Mean [33]	The sum of all pixels divided by the total pixel number
12	Standard Deviation [34]	The measurement of dispersion in the grey intensity level of the image
13	Shannon entropy [35]	The quantity of information present in the ROI images
14	Gray level co-occurrence matrix [36]	The textural information of the ROI regions
15	Skewness [37]	The measure of symmetry in the pixel’s distribution in the image
16	Kurtosis [38]	The density of the pixel’s distribution

**Table 6:** Performance analysis of 11 machine learning models where T_ACC, T_MCC and T_F1 denote the training accuracy, MCC value and F1 score; Te_ACC, Te_MCC and Te_F1 indicate testing accuracy, MCC value and F1 score.

Model	T_ACC(%)	T_MCC (%)	T_F1 score (%)	Te_ACC (%)	Te_MCC (%)	Te_F1(%)	AUC (%)
KNN	100	100	100	92.82	89.27	92.82	95.88
SVC	100	100	100	92.82	75.22	81.99	86.52
DT	100	100	100	94.62	92.46	94.62	96.36
RF	100	100	100	95.91	95.39	95.90	96.74
MLP	70.24	58.43	67.97	70.61	58.80	68.49	82.03
AB	53.05	37.32	53.74	54.39	39.15	55.19	57.12
XBG	99.58	99.44	99.58	95.40	94.97	95.22	96.65
GNB	42.70	23.39	41.04	43.97	25.27	42.34	68.10
SVM	38.01	17.01	34.02	36.96	15.36	32.62	65.72
SGD	40.97	13.73	28.48	40.54	12.42	27.59	57.13
LR	43.06	13.36	42.08	42.90	12.99	41.79	59.91

**Table 7:** Performance analysis of ensemble models where T_ACC, T_MCC and T_F1 denote the training accuracy, MCC value and F1 score, respectively; Te_ACC, Te_MCC and Te_F1 indicate testing accuracy, MCC value and F1 score, respectively.

Model	T_ACC (%)	T_MCC (%)	T_F1 Score (%)	Te_ACC (%)	Te_MCC (%)	Te_F1 Score (%)	AUC (%)
RF-DT-XGB	100	100	100	95.64	95.47	95.64	96.14
RF-XGB	100	100	100	96.57	97.06	96.57	97.30
RF-DT-XGB-SVM-KNN	100	100	100	91.53	88.07	91.39	93.47

**Table 8:** Performance evaluation of different feature selection methods on RF-XGB.

Feature Selection	Configuration	Feature Number	Test ACC (%)	MCC (%)	F1 (%)	AUC (%)
All features	16 features	16	96.03	97.06	96.03	96.50
Univariate Feature	14 features	14	96.58	96.39	96.58	97.54
Univariate Feature	12 features	12	97.35	95.55	97.31	98.21
Correlation Matrix	0.01 threshold	15	96.70	96.56	96.69	97.58
Correlation Matrix	0.015 threshold	14	97.25	96.64	97.25	97.62
Correlation Matrix	0.025 threshold	12	97.35	95.55	97.31	98.21
PAC	-	15	96.70	96.56	96.69	97.58
PAC	-	14	97.25	96.64	97.25	97.62
PAC	-	10	96.74	95.13	96.74	98.03
Wrapper Method	0.05 thresh	14	97.92	96.89	97.92	98.73
Wrapper Method	0.01 thresh	13	97.13	95.72	97.13	98.23
Wrapper Method	0.045 thresh	9	96.74	95.15	96.73	97.97
RF	Threshold 0.25	14	96.90	96.76	96.90	97.48
RF	Threshold 0.045	12	97.81	96.73	97.81	98.67
RF	Threshold 0.05	10	98.05	97.27	98.05	98.91

**Table 9:** Accuracy comparison with existing literature.

Author	Class	No of Images	Method/Model	Accuracy (%)
Meselhy Eltoukhy et al. [8]	2 class: Benign and malignant	322 mammograms	Wavelet coefficient SVM classifier	95.84
Vijayarajeswari et al. [7]	3 class: benign, malignant and normal	95 mammograms	SVM	94.0
Tang et al. [6]	2 class: normal and cancerous	1487 mammograms	voting classification	96.06
***This paper***	4 classes: benign calc, benign mass, malignant calc and malignant mass	1459 mammograms After augmentation: 11,536 images	Geometric feature extraction, Random Forest Feature selection RF-XGB-10 classifier	98.05

## Data Availability

The Cancer Imaging Archive (TCIA) dataset [12] is publicly available.
